# Lineage specific role of *Ship1* in development of allergic airway inflammation

**DOI:** 10.1186/1710-1492-10-S2-A56

**Published:** 2014-12-18

**Authors:** Matthew J Gold, Michael R Hughes, Frann Antignano, Colby Zaph, Kelly M McNagny

**Affiliations:** 1 The Biomedical Research Centre, University of British Columbia, Vancouver, British Columbia, V6T 1Z3, Canada

## Background

The PI3K pathway is a potent mediator of several functions associated with asthma pathogenesis, including supporting leukocyte survival, activation, migration and cytokine release. Proper negative regulation of this pathway is integral in order to restrict overactive immune responses. Negative regulation of PI3K is predominantly controlled by the lipid phosphatases PTEN and SHIP-1. *Inpp5d* (*Ship1*) deficient mice develop spontaneous airway inflammation and have enhanced sensitivity to allergen induced airway inflammation. We hypothesized that deleting *Ship1* expression specifically in lineages known to be crucial for adaptive Th2 responses would uncover more subtle effects that could either positively or negatively regulate disease severity in a mouse model of allergic airway inflammation (AAI).

## Methods

*Ship1* expression was deleted in B cell, T cell and dendritic cell (DC) lineages and the resulting *Ship1*^ΔB cell^, *Ship1*^ΔT^^cell^ and *Ship1*^ΔDC^ mice were exposed to house dust mite (HDM) antigen over an 18-day period. Infiltrating leukocytes in the bronchoalveolar lavage (BAL) and lung, serum antibody levels and Th1 and Th2 cytokine responses were quantified to assess disease severity.

## Results

Deletion of *Ship1* in either the B cell, T cell or DC lineages did not result in spontaneous airway inflammation, and loss of *Ship1* in the B cell linage did not affect HDM-induced AAI. Surprisingly, loss of *Ship1* in either of the T cell or DC lineages protected from development of AAI by skewing the HDM-induced immune response to a Th1 phenotype instead of the characteristic Th2 phenotype associated with allergic asthma.

## Conclusions

While loss of *Ship1* expression throughout the hematopoietic populations leads to spontaneous lung inflammation, selective deletion of *Ship1* in T cells and DCs impairs the formation of an adaptive Th2 response and protects from the development of AAI.

